# Evaluation of the German Version of the Adult Attention-Deficit/Hyperactivity Disorder Self-Report Screening Scale for DSM-5 as a Screening Tool for Adult Attention-Deficit/Hyperactivity Disorder in Primary Care

**DOI:** 10.3389/fpsyg.2022.858147

**Published:** 2022-04-22

**Authors:** Cora Ballmann, Markus Alexander Kölle, Ines Bekavac-Günther, Florian Wolf, Florian Pargent, Anne Barzel, Alexandra Philipsen, Jochen Gensichen

**Affiliations:** ^1^Institute of General Practice and Family Medicine, University Hospital of the Ludwig-Maximilians-University, Munich, Germany; ^2^Department of Psychiatry and Psychotherapy, University Hospital Bonn, Bonn, Germany; ^3^Institute of General Medicine, Ulm University, Ulm, Germany; ^4^Institute of General Practice and Family Medicine, Jena University Hospital, Jena, Germany; ^5^Department of Psychology, LMU Munich, Munich, Germany

**Keywords:** ASRS-5, ADHD, screening, primary care, DSM-5, adult

## Abstract

Adult attention-deficit/hyperactivity disorder (ADHD) is common, but often undiagnosed. A valid and time-efficient screening tool for primary care is needed. Objective of this study is to evaluate the German version of the Adult ADHD Self-Report Scale for DSM-5 (ASRS-5) and its feasibility, acceptability, and reliability as a screening tool for adult ADHD in primary care. A multi-centered prospective, diagnostic study was performed. We recruited 262 patients in primary care practices and at an ADHD Outpatient Service of a department of psychiatry in Germany. Patients from 18 to 65 years with suspected or diagnosed ADHD were included by medical doctors, as well as non-ADHD patients as “negative controls.” Participants filled in the ASRS-5 and a sociodemographic questionnaire. The Integrated Diagnosis of Adult ADHD, revised version (IDA-R) performed by trained interviewers was used for validation. Feasibility, acceptability, and credibility in primary care practices were examined through a semi-structured interview. The German version of the ASRS-5 showed comparable psychometric properties to the English original version (sensitivity 95.6% and specificity 72.3%). For factor structure, a parallel analysis suggested one latent dimension. Performing confirmatory factor analysis, the best fit was achieved for a general factor with one correlated error. Internal consistency results in Raykovs Omega = 0.86 and Cronbach’s *α* = 0.88. The ASRS-5 was assessed positively in terms of feasibility, acceptability, and credibility by interviewed general practitioners. Potential problems were raised for “treatment options,” “stigmatization,” and “knowledge gaps.” In conclusion, the German version of the ASRS-5 offers a promising tool to improve adult ADHD patients’ diagnosis and healthcare.

## Introduction

Adult attention-deficit/hyperactivity disorder (ADHD) is common. In at least half of the children affected by ADHD, symptoms persist until adulthood (Sibley et al., 2017, 2021). Adult patients often predominantly suffer from attention deficit rather than the well-known and conspicuous ADHD features of hyperactivity/impulsivity (Biederman et al., 1996, 2000; Karam et al., 2015). The wording of the DSM diagnosis criteria intitally has been set up to the typical ADHD symptoms in children. Nowadays, it is known that adult ADHD includes further aspects, such as executive dysfunction, emotion regulation, and disorganization (Barkley, 1997; Wender, 1998; Conners and Sparrow, 1999). These all lead to impairment in everyday life. Moreover, psychiatric comorbidities, such as depression, anxiety, substance use disorder, social phobia, and others, are common (Kessler et al., 2006; Fayyad et al., 2017). While the prevalence of ADHD in adults in Germany is estimated at around 3%, health insurance data show a diagnosis frequency of only 0.2%–0.4% (de Zwaan et al., 2012; Bachmann et al., 2017; Fayyad et al., 2017). One reason may be the fragmentation of ADHD. During the transition from adolescence to adulthood a lot of patients drop-out from services (Robb and Findling, 2013; Eklund et al., 2016). Moreover, comorbidities might cover ADHD diagnosis. Without diagnosis, patients do not have access to evidence-based treatment, which is known to be effective. In the German healthcare system as well as in others, general practitioners (GP) play an important role as gatekeepers (French et al., 2020). One of their roles is to identify patients initially. Guidelines therefore emphasize an urgent need for a valid and time-efficient screening tool for adult ADHD in primary care [Deutsche Gesellschaft für Psychiatrie und Psychotherapie PuND, Deutsche Gesellschaft für Suchtforschung und Suchttherapie e.V. (DG-SUCHT), 2020].

The World Health Organization (WHO) Adult ADHD Self-Report Screening Scale for DSM-5 (ASRS-5) is a six-item questionnaire (Ustun et al., 2017). In contrast to previous screening tools, it refers to the current DSM-5 criteria (Ustun et al., 2017). The original English ASRS-5 version promises good psychometric properties (sensitivity 91.4%, specificity 96% in a general population sample; sensitivity 91.9%, specificity 74% in a clinical sample; Ustun et al., 2017). Because it is time-efficient and empirically validated, the English ASRS-5 meets the criteria for a capable screening tool.

The objective of this study is to evaluate the psychometric properties of the German version of the ASRS-5, its feasibility, acceptability, and credibility as a screening tool for adult ADHD in primary care.

## Materials and Methods

### Translation

Translating the English ASRS-5 into German was performed according to the WHO Composite International Diagnostic Interview (CIDI) translation guideline. The developer approved the final translation after receiving report of the requested information about the process (parallel translations with back translation, expert panel review, pretesting, and cognitive interviewing; Sousa and Rojjanasrirat, 2011). The German version of the ASRS-5 is available at https://www.hcp.med.harvard.edu.

### Aim of the Current Study and Study Design

For this study, we had two hypotheses: (1) The German translation of the ASRS-5 shows similar psychometric properties (sensitivity, specificity) as the English original and therefore is a valid screening tool for adult ADHD; (2) The ASRS-5 is a feasible, accepted, and credible screening tool for adult ADHD in primary care and thus might be implemented well in everyday practice.

Therefore, we performed a multi-centered, prospective, diagnostic study, and we examined a convenience sample of 262 patients. Patients were recruited in GP practices in South and West Germany and at the ADHD Outpatient Service of the department of psychiatry and psychotherapy at the University of Bonn. Medical doctors did the eligibility check based on the file (ICD-10 diagnosis, documentation of medication) and included the patients. Patients filled in the ASRS-5 in paper–pencil form. To check external validity, trained interviewers validated the diagnosis by phone or in person, using the revised version of the Integrated Diagnosis of Adult ADHD (IDA-R) a semi-structured DSM-5 interview. For internal consistency, we determined Cronbach’s *α*. To evaluate the feasibility, acceptability, and credibility of the ASRS-5 as a screening tool for adult ADHD in primary care, a semi-structured phone interview was conducted with the participating GPs.

### Study Population

Patients aged 18–65 years with suspected or diagnosed ADHD (F90.0, F98.8) according to guidelines [Deutsche Gesellschaft für Psychiatrie und Psychotherapie PuND, Deutsche Gesellschaft für Suchtforschung und Suchttherapie e.V. (DG-SUCHT), 2020] or intake of methylphenidate or atomoxetine were included. Patients with common comorbidities like mild to moderate depression (F32.0/1, F33.0/1/4), antidepressant monotherapy, anxiety (F41.0/1/2), social phobia (F40.1), or substance use disorder (F10.1/2, F12.1/2) were included due to frequent occurrence to ensure the representativeness of the investigated patient group. As negative controls, patients were included who show up in the primary care practice for a different concern and do not satisfy any of the criteria above. Patients with a differential diagnosis of schizophrenia as well as schizotypal and delusional disorders (F20-29), intake of antipsychotic medication, bipolar disorder (F31), mania (F30), emotional instable personality disorder (F60.3), and severe depression (F32.3, F33.3) were excluded from the study.

### Adult Attention-Deficit/Hyperactivity Disorder Self-Report Scale for DSM-5

The WHO developed the ASRS-5 based on the currently valid DSM-5 criteria. For the development of the ASRS-5, a novel machine learning algorithm called RiskSLIM (Ustun and Rudin, 2019) was used. RiskSLIM constructs simple and interpretable risk scores that are optimized for predictive performance while satisfying practical constraints (e.g., small number of items, test score based on the sum of small integer values, and optional monotonicity constraints; Ustun and Rudin, 2019). RiskSLIM empirically reduced a larger item pool resulting in six items asking for DSM-5 symptoms of inattention (*n* = 1), non-DSM-5 symptoms of executive dysfunction (*n* = 2), and DSM-5 symptoms of hyperactivity and impulsivity (*n* = 3) with a five-point Likert scale (never/rarely/sometimes/often/very often). Patients are supposed to indicate the frequency of the queried behaviors, feelings and thoughts during the last 6 months. For scoring the ASRS-5, the developers proposed an evaluation scheme with a cutoff of ≥14 for a positive screening result. In this scheme, answer categories for questions were weighted differently. A sum score is built. If this is ≥14, the ASRS-5 is positive. The English original version of the ASRS-5 was validated within a mixed cohort of 637 participants where it showed good psychometric properties (see above; Ustun et al., 2017).

### Integrated Diagnosis of Adult ADHD, Revised Version

The IDA-R is a standardized, established German diagnostic guide (Retz et al., 2013; Wolfgang Retz and Rösler, 2018). It consists of three validated diagnostic tools which were carried out with each participant. The first tool is the ASRS-V1.1, the precursor of the ASRS-5, which also consists of six items with a five-point Likert scale (Kessler et al., 2005). In contrast to the ASRS-5, the ASRS-V1.1 is based on the DSM-4 criteria (Kessler et al., 2005). If four or more responses satisfy the evaluation template (ASRS-V1.1 score ≥ 4), the screening is positive (Kessler et al., 2005). Studies showed a sensitivity of 68.7% and a specificity of 99.5% for the English version (Adler et al., 2006). A validation of the German version within a Swiss cohort yielded a sensitivity of 66.6% and a specificity of 64.9% (Buchli-Kammermann et al., 2011). In a second step, existence of childhood symptoms was evaluated retrospectively, using a validated adaption of the German short version of the Wender Utah rating scale (WURS-k; Retz-Junginger et al., 2002; Retz et al., 2013). A WURS-k score ≥ 6 rates positive (Retz-Junginger et al., 2002; Retz et al., 2013). Validation studies showed a sensitivity of 91% and specificity of 85% for this cutoff (Retz-Junginger et al., 2002). Lastly, a semi-structured DSM-5 interview was conducted to check whether the ADHD symptoms meet the diagnostic criteria according to DSM-5 (Retz et al., 2013; Wolfgang Retz and Rösler, 2018). The validity and reliability of this interview were evaluated based on interviews with 147 patients of the ADHD consultation hour of the university hospital Homburg in a previous study (Retz et al., 2013). Here, internal consistency was 0.87 (Cronbach’s *α*; Retz et al., 2013). External validity was verified using Adult ADHD Self-Report Scale (correlation coefficient 0.66) and Wender-Reimherr Interview (correlation coefficient 0.77; Retz et al., 2013).

### Process Evaluation

Setting a focus on process evaluation in general practice, a secondary aim of this study was to evaluate the feasibility, acceptability, and credibility of the ASRS-5. Its application as a screening tool for adult ADHD in primary care was defined to be the target behavior according to the Theoretical Domains Framework (TDF). For examination of possible influences on implementation, we chose a Determinant Framework. Here, we used the Consolidated Framework for Implementation Research (CFIR; Damschroder et al., 2009). The CFIR consists of the following six domains: intervention characteristics, outer setting, inner setting, characteristics of the individual, and process (Damschroder et al., 2009). Using the CFIR Interview Guide Tool^1^, a semi-structured interview was set up. Each domain was represented by one question. The interviews were conducted with participating general practitioners and held by phone or in person. All interviews were recorded, transcribed verbatim, and a structured content analysis was performed according to the Mayring method (Mayring, 2000).

### Data Analysis

Sensitivity and specificity were calculated with SPSS 26. Power analysis, Wilson confidence intervals (package DescTools; Signorell et al., 2021), Raykovs Omega (package SemTools; Jorgensen et al., 2021), and Cronbach’s *α* were computed in R. Exploratory Maximum Likelihood (ML) factor analyses and ML parallel analysis (package psych; Revelle, 2017; level of significance = 0.05; 2,000 resampling iterations; communalities estimated by squared multiple correlation) as well as ML confirmatory factor analysis (package lavaan; Rosseel, 2011; default settings) were performed. ML estimation was chosen because exploratory factor analysis for categorical data (using polychoric correlations) could not be estimated as some extreme response categories of the ASRS-5 items were not selected by our sample.

### Calculation of Power and Sample Size

Sample size calculation [R package DescTools (Signorell et al., 2021)] was performed based on the following assumptions:

- For a positive screening, the original cutoff from the English ASRS-5 version is used.
- A two-sided binomial hypothesis test (Level of significance = 0.05) is performed with the null hypothesis that the sensitivity is exactly 0.9 (based on the English version).
- The true sensitivity of the German version is equal to 0.8 (0.1 as the minimal deviation of interest which should be recognized by the test).
- The number of participants with a positive diagnosis in IDA-R is 100.

Under these assumptions, the power of this hypothesis test is approx. 0.8. Calculating a confidence interval for the true sensitivity of the German version, the expected length of the confidence interval would be approx. 0.15.

### Ethics

The Ethics Committee of the University Hospital, LMU Munich (No. 20-0882) approved the study. Informed written consent was obtained from all individuals who volunteered to participate in the study. General practitioners received 20 € for each included patient.

## Results

### Cohort

Complete data from 262 patients were obtained for final analysis. Patients were recruited in 23 general practices (Munich *n* = 16, Ulm *n* = 7). Each general practice contributed 1–28 patients to the study. Moreover, 91 patients were recruited at the ADHD Outpatients Service in Bonn. The patients’ mean age was 39 (*SD* 13.6, 18–65) years. Around 118 (45%) patients were male, 143 (55%) were female, one was non-binary. Educational level was high with 58.8% having at least A levels. A total of 96 (36%) patients indicated previous mental disorder, 55 (21%) declared the intake of psychotropic medication during the last 4 weeks. For complete patient data, see Table 1.

**Table 1:** Clinical data.

	Total	GP setting	ADHD consultation hour
**Socio-demographics**			
Gender	262	171	91
Male	118 (45)	94 (55)	49 (53.8)
Female	143 (54.6)	77 (45)	41 (45.1)
Divers	1 (0.4)	0	1 (1.1)
Missing values	0	0	0
Age	Mean 39 (SD 13.6, 18–65)	Mean 41 (SD 13.9, 18–65)	Mean 36 (SD 12.3, 18–61)
**Level of education**	262 years	171 years	91 years
≥ Highschool	100 (38.2)	77 (45.0)	23 (25.3)
≥ A Levels	154 (58.8)	90 (52.6)	64 (70.3)
Others	6 (2.3)	2 (1.2)	4 (4.4)
Missing values	2 (0.8)	2 (1.2)	0
**Clinical variables**			
*Previous mental illness*	262	171	91
No	153 (58)	117 (68.4)	36 (39.6)
Yes	96 (36)	43 (24.6)	54 (59.3)
Missing values	13 (5)	12 (7)	1 (1.1)
Depression	80	35	45
Anxiety	80	16	64
Social phobia	80	7	73
Substance use disorder	93	14	79
**Any psychotropic medication (last 4 weeks)**	262	171	91
Yes	55 (21.0)	22 (12.9)	33 (36.3)
No	202 (77.1)	146 (85.4)	56 (61.5)
Missing values	5 (2.0)	3 (1.8)	2 (2.2)
**ADHD severity (semi-structured interview)**			
Low (5–9 symptoms)	64	21	43
Moderate (10–11 symptoms)	32	13	21
High (12–14 symptoms)	28	9	19
Very high (15–18 symptoms)	8	2	6
Missing values	0	0	0
**ADHD presentation**	113		
Inattentive	64	13	51
Hyperactive	11	6	5
Combined	38	15	23
Missing values	0	0	0

SD, standard deviation.

### ASRS-5 Screening

A total of 150 (57.3%) participants were screened positive using the ASRS-5 (62 in GPs, 88 in ADHD consultation hour). Of these, 67 (44.7%) were female and the mean age was 36 (*SD* 12.6, 18–64) years. ASRS-5 positive results correlated significantly with previous mental disorder, previous psychotherapy, intake of psychotropic medication during the last 4 weeks (Phi/Cramer-V test *p* < 0.01), as well as with ADHD severity (Chi^2^ test *p* < 0.01) and age (Eta coefficient *p* < 0.01). There was no significant correlation with sex, and educational level (Phi/Cramer-V test). A total of 113 participants had a positive validation (IDA-R). In this group, 52 (46%) were female and the mean age was 36 (*SD* 12.2, 18–61) years. A positive IDA-R correlated significantly with the same parameters like a positive ASRS-5 [previous mental disorder, previous psychotherapy, and intake of psychotropic medication during the last 4 weeks (Phi/Cramer-V test *p* > 0.01), ADHD severity (Chi^2^ test *p* < 0.01) and age (Eta coefficient *p* < 0.01)]. Here, again there was no significant correlation with sex and educational level (Phi/Cramer-V test).

### Factor Structure

Parallel analysis suggested a one-dimensional structure but confirmatory factor analysis did not confirm this $(\chi_{df=9}^{2}=37.372,p=0.000,RMSEA=0.110,CFI=0,963,SRMR=0.032).$ Checking residual correlations, the best fit was achieved for a general factor with one correlated error $\left(\chi_{df=8}^{2}=4.967,p=0.761,RMSEA=0.000,CFI=1.000,SRMR=0.014\right)$ between items 5 and 6 (0.401). Using this model, reliability was estimated with the internal consistency measures by Raykovs Omega (Raykov, 2001; 0.86) and Cronbach’s *α* (0.88). EzCutoff (Schmalbach et al., 2019) suggested that appropriate cutoffs for fit indices are CFI = 0.989, RMSEA = 0.065, and SRMR = 0.026 for our specific model and sample size. The empirical fit values fulfill these criteria.

### Psychometric Properties

The German version of the ASRS-5 showed comparable psychometric properties to the English original version. Sensitivity was very high (95.6%; Wilson CI [0.90, 0.98]), while specificity was a bit lower (72.3%; 95% Wilson CI [0.65, 0.79]; Table 2). For male respondents (*n* = 142), psychometric properties were 95% (sensitivity) and 70.7% (specificity). For female respondents (*n* = 118), psychometric properties were 96.2% (sensitivity) and 74.2% (specificity). Male and female respondents did not differ significantly in age (*t*-test), educational level, previous mental illness, ADHD severity, and ADHD subtype (Mann–Whitney U test).

**Table 2:** Sensitivity and specificity.

	IDA-R pos	IDA-R neg	Total
ASRS 5 pos	108	41	149
ASRS 5 neg	5	107	112
Total	113	148	261
	Sensitivity 108/113 = 95.6% 95% Wilson CI [0.90, 0.98]	Specificity 107/148 = 72.3% 95% Wilson CI [0.65, 0.79]	

### ASRS-5 vs. ASRS-V1.1

For the ASRS-V1.1 as part of the IDA-R, the sensitivity of only 87.6% was lower than for the ASRS-5. Specificity (85.1%) of the ASRS-V1.1 was a bit higher.

### Process Evaluation

Around 11 general practitioners were interviewed for the evaluation of the feasibility, acceptability, and credibility of the ASRS-5 as a screening tool for adult ADHD in primary care. Overall, the feedback was very positive. Being short and comprehensible, the ASRS-5 demonstrated good feasibility. The majority indicated that they would use the screening tool in future and would discuss it with others, indicating high acceptability. Positive feedback, especially from ADHD patients lends credibility to the ASRS-5. Stigmatization, accessibility of further specialist care, and knowledge gaps for ADHD appeared as potential problems in the inductive content analysis.

## Discussion

The objective of this study was to evaluate the psychometric properties of the German version of the ASRS-5, its feasibility, acceptability, and credibility as a screening tool for adult ADHD in primary care.

### Current Study

Here, we examined a convenience sample of 262 patients, which were recruited in general practices and an ADHD Outpatients Service. Of these 150 were screened positive (ASRS-5) and 113 had a positive validation (IDA-R). Psychometric properties showed 95.6% sensitivity and 72.3% specificity. It was the first time the German version of the ASRS-5 was evaluated. Our study differed from others by investigating in the primary care setting with its particular conditions. Process evaluation revealed a positive feedback from general practitioners about the ASRS-5.

The developers of the ASRS-5 assessed a total of 637 participants within three cohorts. One was recruited through population survey (*n* = 119), the others were a managed care (*n* = 218) and a clinical sample (*n* = 300). The overall psychometric properties were well (sensitivity 91.4%, specificity 96%). For the clinical sample with a much higher ADHD prevalence, the specificity was a bit lower (74%), while sensitivity (91.9%) being similar. Our results interestingly are comparable to those of the clinical sample. Sociodemographic data in the original study were only specified for respondents of the overall cohort who met the DSM-5 criteria for adult ADHD. The mean age was 33.1 years, which is similar to the IDA-R positive patients of our cohort (mean age 35.7 years). Both cohorts differ in gender proportion (45.9 vs 53.1% male). Educational level and further clinical criteria were not indicated in the original study (Ustun et al., 2017).

Baggio et al. translated the ASRS-5 to French. They investigated in a sample consisting of outpatients with ADHD, which they subdivided in patients with (*n* = 36) or without bipolar disorder or borderline disorder (*n* = 236), and 285 adults without ADHD who were mainly healthy volunteers (*n* = 248). They measured lower sensitivity (84.3%) and a higher specificity (91.9%) for outpatients without comorbidities than we did for our cohort. Their cohort was similar to ours concerning sex (55.7 vs. 55% female), age (37.9 ± 14.7 vs. 39 ± 13.6 years), and intake of psychotropic medication (20.9 vs. 21%). While we did not see a significant correlation between educational level and positive ASRS-5/IDA-R, they report a lower educational level in ADHD patients. Their study moreover differentiates from ours by determining a new cutoff instead of using the one proposed by Ustun et al. (2017), Baggio et al., (2021).

Somma et al. evaluated the Italian version of the ASRS-5. Their cohort was quite different including 564 only male adolescents (mean age 15.5 ± 1.6 years). Their sensitivity (75%) as well as specificity (65%) using the same evaluation scheme and cutoff as we did (cutoff  ≥ 14), were both way lower than in our study. This might be because of differences in the cohorts. Younger and male patients may differ in symptom manifestation. Somma et al. also performed factor structure analysis which in contrast to our analysis strongly supports a unidimensional structure of the ASRS-5 (Somma et al., 2021).

Genç et al. examined a convenience sample consisting of 68 patients receiving an ADHD diagnosis after applying at psychiatry outpatient clinics and 68 control patients applying to outpatient clinics other than psychiatry and do not have previous ADHD diagnosis. They were using the Turkish version of the ASRS-5. ADHD patients in their cohort were younger (mean age 29.22 ± 8.80 vs. 36 ± 12.2 years), while gender proportions were quite similar (54.4 vs. 55% female). Educational level was also very high (69.1% University), while we used a different measure (58.8% A levels). They did not asses comorbidities and intake of psychotropic medicine. The calculated cutoff of ≥10 revealed a bit lower sensitivity (85.1 vs. 95.6%) and a higher specificity (89.5 vs. 72.3%) than we do. It remains unclear which evaluation scheme weighting of the item was used. The difference in psychometric properties may reveal to a smaller cohort, younger age, or maybe existing comorbidities which unfortunately were not indicated (Genç et al., 2021).

Moreover, note that in our cohort almost 60% of participants had a high educational level which is a significantly higher proportion in comparison to the standard population. Interestingly, we cannot find a significant correlation between educational level and positive ASRS-5/IDA-R. The most common ADHD presentation for our sample was the predominantly inattentive.

### Factor Analysis

While parallel analysis (which can be insensitive to correlated errors) suggested one factor, confirmatory factor analysis revealed a correlated error between items 5 and 6 (i5: How often do you put things off until the last minute?, i6: How often do you depend on others to keep your life in order and attend to details?). Thus, it seems that the last two items of the ASRS-5 share some content beyond the general ADHD factor. Both items ask for non-DSM-5 symptoms of executive dysfunction and we suspect that responses to both items are also affected by personality traits like conscientiousness. This correlated error should be replicated with a new data set. The fit of the one-dimensional model with correlated error was adequate. This result is not trivial because the original scale was constructed with a machine learning algorithm that optimizes predictive performance but does not consider dimensional structure in any way. Our results suggest that despite the predictive focus, the psychometric properties of the ASRS-5 are comparable to more conventional scales.

### Process Evaluation

General practitioners see a lot of patients each day within the whole range of diseases. For this setting, potentially even more than for others, feasibility aspects like processing time matter. The ASRS-5 was assessed positively in terms of feasibility, acceptability, and credibility by interviewed general practitioners. Upcoming topics were further treatment options, stigmatization, and knowledge gaps, all which might be connected. A brief primary care intervention combines knowledge about adult ADHD and a treatment option (Ballmann et al., 2011). This in turn might counteract stigmatization. Further studies should investigate this approach.

### Implications

For this study, we choose the ASRS-5 as it showed good psychometric properties before and seems to be pragmatic for the primary care setting. We could confirm both of this with our results. Nevertheless, a specificity of 72.3% means, there is a risk especially of false positive respondents. This is why we want to underline the ASRS-5 is not an instrument for a definitive diagnosis of adult ADHD. It can be used as a pragmatic tool to confirm or reject an initial suspicion of the treating practitioner, which is the particular need for the primary care setting. If a patient is screened positive further evaluation is needed. According to the German guideline, a diagnosis needs to be confirmed by a specialist [Deutsche Gesellschaft für Psychiatrie und Psychotherapie PuND, Deutsche Gesellschaft für Suchtforschung und Suchttherapie e.V. (DG-SUCHT), 2020]. However, appointments are rare and patients often have to wait for a long time (Kammer, 2011; Bachmann et al., 2017). Moreover, a stepped care model with a broader scope of action for general practitioners might solve this problem. Therefore, a structured diagnostic recommendation would be useful.

### Limitations

Our study examined a convenience sample collected both at GP practices and at an outpatient clinic specialized in ADHD patients. The high base rate of ADHD in our study population (the number of patients with a positive IDA-R was even higher than assumed for our sample size calculation) is not representative for GP settings in which the ASRS-5 scale will be mostly applied. Thus, we do not report Positive and Negative Predictive Value which both depend on the base rate of ADHD in the studied population. Additionally, we want to caution GPs against overconfidence when applying the ASRS-5 as a screening tool in primary care. For the sake of argument, assume a general prevalence of ADHD in German adults of 3% (Fayyad et al., 2017) in combination with the sensitivity (95.6%) and specificity (72.3%) estimates from our sample. Simple calculations (Labarge et al., 2003) reveal that while more than 99.5% of clients with a negative screening result in the ASRS-5 would be expected to not suffer from ADHD (Negative Predictive Value), less than 10% of clients with a positive screening result would be expected to actually suffer from ADHD (Positive Predictive Value).

In our study, we focused on sensitivity and specificity which do not directly depend on the base rate of ADHD. However, sensitivity and specificity can vary between populations that not only differ with respect to the base rate but also with respect to other person characteristics that affect the functional relationship between the ASRS-5 scale and a true ADHD diagnosis. Such an effect can be seen in the original publication where specificity was noticeably lower in the clinical sample (74 vs. 96%) that was collected in a slightly different setting (Ustun et al., 2017). Wide inclusion criteria especially in terms of comorbidities probably had an impact on our results. Here, this is intended because those are representative for the primary care setting.

In this study we choose the ASRS-5 as it showed good psychometric properties before and seems to be pragmatic for the primary care setting. Please note that some points of the ASRS-5 can be seen critically. It is based on DSM-5, but the wording is quite different. Besides, two out of six items relate to non-DSM-5 symptoms of executive dysfunction. Moreover only one item relates to DSM-5 symptom of inattention which is known to often be predominant in adults with ADHD (Ustun et al., 2017).

For validation, we used the IDA-R which consists of the ASRS-V1.1, the WURS-k, and a semi-structured DSM-5 interview. It can be criticized that ASRS-V1.1 refers to DSM-4, while the examined ASRS-5 refers to DSM-5. Furthermore, the WURS-k assesses childhood ADHD symptoms by retrospective self-report. References show, that this might not be reliable (Mannuzza et al., 2002; Retz-Junginger et al., 2002). One more limitation is that for logistic reasons, interviewers for semi-structured DSM-5 interviews were not blinded.

Regarding factor analysis, note that polychoric correlations or estimators for discrete ordered responses categories could not be used because response categories were not fully utilized. The choice of response categories could be therefore reconsidered.

In this study, we used the evaluation scheme proposed from Ustun et al. (2017) with a cutoff ≥ 14. Because of the limited sample size, we did not perform ROC analysis to determine a new cutoff for the German translation. Thus, we accept the specificity of 72.3% for the benefit of high sensitivity which is the most important value for a screening tool.

## Conclusion

A time-efficient screening tool for adult ADHD validated within the primary care setting may help to improve quality of care. The ASRS-5 meets the criteria of a capable screening tool. Here, we reported positive evidence with regard to the psychometric properties of the German version of the ASRS-5, as well as to its feasibility, acceptability, and credibility in the primary care setting. The German version of the ASRS-5 offers a promising tool to improve adult ADHD patients’ diagnosis and healthcare.

## Data Availability Statement

The raw data supporting the conclusions of this article will be made available by the authors, without undue reservation.

## Ethics Statement

The Ethics Committee of the University Hospital, LMU Munich (No. 20-0882) approved the study. The patients/participants provided their written informed consent to participate in this study.

## Author Contributions

CB: designed and delivered the study and wrote the manuscript. MK and IB-G: delivered the study. FW: contributed to the study design, delivered the study, and contributed to the final manuscript. FP: did the statistics and contributed to the final manuscript. AB and AP: contributed to the study design and final manuscript. JG: designed the study and contributed to the final manuscript. All authors contributed to the article and approved the submitted version.

## Funding

This study was funded by Stiftung Allgemeinmedizin (The Primary Health Care Foundation) and intramural funding (University of Bonn).

## Conflict of Interest

AP: advisory activity for Shire/Takeda, Medice, Boehringer, Janssen-Cilag; Lecture fees from Medice, Takeda; research funding through Medice; behavioral therapy supervisor and DBT supervisor; and author of book articles and books on ADHD.

The remaining authors declare that the research was conducted in the absence of any commercial or financial relationships that could be construed as a potential conflict of interest.

## Publisher’s Note

All claims expressed in this article are solely those of the authors and do not necessarily represent those of their affiliated organizations, or those of the publisher, the editors and the reviewers. Any product that may be evaluated in this article, or claim that may be made by its manufacturer, is not guaranteed or endorsed by the publisher.

## Footnotes

^1^ https://cfirguide.org/guide/app/#/
