# Supplementary material for: Evaluation of the German Version of the Adult Attention-Deficit/Hyperactivity Disorder Self-Report Screening Scale for DSM-5 as a Screening Tool for Adult Attention-Deficit/Hyperactivity Disorder in Primary Care
Source: Front Psychol. 2022 Apr 22;13:858147. doi: 10.3389/fpsyg.2022.858147 (PMC9075696; doi:10.3389/fpsyg.2022.858147)
Supplement: Supplementary file 1 [file Table_1.DOCX]

Supplementary Data

**Calculation of Power and Sample Size**

library(DescTools)

set.seed(1)

B = 100000

# power for two.sided binomial test with

# H0: sensitivity != H0_sensitivity, true sensitivity = true_sensitivity

# alpha = 0.05, number of truly sick people = n_sick

sim_power = function(n_sick, H0_sensitivity, true_sensitivity){

test = binom.test(rbinom(1, size = n_sick, prob = true_sensitivity),

n = n_sick, p = H0_sensitivity, alternative = "two.sided")

test$p.value < 0.05

}

H0_sensitivity = 0.9

true_sensitivity = 0.8

n_sick = 100

tests = replicate(B, sim_power(n = n_sick, H0_sensitivity = H0_sensitivity, true_sensitivity = true_sensitivity))

mean(tests) # power

# [1] 0.80601

# expected CI width for two.sided wilson CI with

# true sensitivity = true_sensitivity, number of truly sick people = n_sick

sim_CI_width = function(true_sensitivity, n_sick){

ci = BinomCI(rbinom(1, size = n_sick, prob = true_sensitivity), n = n_sick, method = "wilson")

ci[,"upr.ci"] - ci[,"lwr.ci"]

}

true_sensitivity = 0.8

n_sick = 100

CI_width = replicate(B, sim_CI_width(n_sick = 100, true_sensitivity = true_sensitivity))

mean(CI_width) # expected CI width

# [1] 0.1543177

**Wilson Confidence Intervals**

library(DescTools)

# Confidence intervals for conference presentation (partial data)

## sensitivity

BinomCI(105, n = 108, conf.level = 0.95, method = "wilson")

# est lwr.ci upr.ci

# [1,] 0.9722222 0.9214966 0.9905087

## specificity

BinomCI(48, n = 76, conf.level = 0.95, method = "wilson")

# est lwr.ci upr.ci

# [1,] 0.6315789 0.5192507 0.7312458

## positive predictive value

BinomCI(105, n = 135, conf.level = 0.95, method = "wilson")

# est lwr.ci upr.ci

# [1,] 0.7777778 0.7005136 0.8396708

# Confidence intervals for paper publication (full data)

## sensitivity

BinomCI(108, n = 113, conf.level = 0.95, method = "wilson")

# est lwr.ci upr.ci

# [1,] 0.9557522 0.9005823 0.9809541

## specificity

BinomCI(107, n = 148, conf.level = 0.95, method = "wilson")

# est lwr.ci upr.ci

# [1,] 0.722973 0.645926 0.7887379

## positive predictive value

BinomCI(108, n = 149, conf.level = 0.95, method = "wilson")

# est lwr.ci upr.ci

# [1,] 0.7248322 0.6481543 0.7902084

**Factor anyalysis**

> CORA <- read.spss("~/desktop/R_Daten/cora.sav", to.data.frame = TRUE)

> list(CORA)

[[1]]

i1 i2 i3 i4 i5 i6

1 1 2 3 4 4 1

2 0 0 3 0 2 0

3 1 3 4 2 2 1

4 1 1 1 2 4 3

5 4 0 4 3 4 2

6 4 1 4 4 4 4

7 3 4 1 3 4 2

8 3 0 2 2 4 3

9 1 0 1 1 0 0

10 1 1 0 1 2 0

11 0 0 2 1 1 0

12 2 1 3 1 1 2

13 3 3 4 3 4 3

14 3 0 4 2 2 3

15 1 0 2 4 4 3

16 2 1 3 4 4 2

17 3 3 4 4 4 2

18 1 2 3 2 1 1

19 1 1 2 2 3 1

20 2 1 3 4 3 1

21 1 0 3 3 4 2

22 0 0 1 1 0 0

23 3 0 2 2 3 2

24 1 0 3 2 3 2

25 2 0 3 2 3 3

26 0 0 0 2 2 0

27 0 0 0 0 0 0

28 1 1 2 2 1 0

29 4 3 4 3 3 3

30 1 0 1 1 2 0

31 0 0 0 0 0 0

32 0 0 0 2 2 0

33 1 0 1 1 1 1

34 0 0 0 1 0 0

35 0 0 1 2 1 0

36 4 2 4 0 3 0

37 1 1 2 1 2 0

38 0 0 1 1 2 0

39 3 2 4 3 4 2

40 4 2 4 3 4 3

41 1 0 3 2 2 0

42 4 2 5 4 5 5

43 2 0 1 3 2 0

44 1 0 0 0 1 0

45 1 0 2 2 3 2

46 1 0 2 1 2 1

47 1 0 2 0 2 0

48 2 2 3 3 4 3

49 2 0 1 3 4 2

50 0 0 2 1 0 1

51 0 0 2 3 1 0

52 0 0 0 0 2 0

53 1 0 0 1 2 1

54 2 2 3 2 1 0

55 1 0 2 2 2 1

56 1 0 1 2 2 1

57 1 0 1 0 0 0

58 1 0 2 0 3 0

59 0 0 0 1 2 0

60 1 0 2 1 2 1

61 3 1 3 4 2 2

62 1 2 2 1 0 2

63 2 0 3 2 2 4

64 1 0 3 4 3 2

65 0 0 1 0 0 0

66 1 0 2 1 2 0

67 2 1 3 2 3 2

68 1 0 2 0 4 2

69 3 1 4 0 4 3

70 2 0 4 1 2 0

71 2 0 2 0 0 1

72 1 0 3 0 4 4

73 0 0 3 0 0 0

74 0 0 3 1 3 1

75 2 1 3 3 2 1

76 0 0 0 1 1 0

77 1 0 0 0 0 0

78 0 0 0 0 0 0

79 2 1 3 1 1 1

80 0 0 0 2 2 0

81 0 0 0 0 0 0

82 0 0 1 1 1 1

83 1 0 1 1 2 0

84 1 0 3 1 3 0

85 1 0 2 2 1 0

86 1 0 2 0 0 0

87 0 0 1 2 0 0

88 0 0 2 1 3 1

89 0 0 1 1 1 0

90 2 1 1 1 3 2

91 2 2 4 3 3 2

92 4 3 3 3 3 1

93 2 0 3 4 0 1

94 1 0 0 0 3 0

95 0 0 0 0 3 2

96 2 0 1 2 2 1

97 4 3 4 4 3 3

98 4 0 2 0 4 3

99 4 2 2 4 4 3

100 3 2 2 4 4 3

101 4 0 4 1 2 4

102 3 2 3 4 4 4

103 4 1 4 4 4 3

104 4 3 4 4 4 4

105 3 0 2 1 4 4

106 3 2 2 4 4 4

107 3 1 3 4 4 4

108 4 3 4 4 3 4

109 4 4 4 0 4 4

110 3 2 3 4 3 2

111 2 2 4 3 4 2

112 4 2 3 4 3 2

113 4 2 1 3 4 3

114 3 0 4 4 4 3

115 3 3 4 3 4 3

116 2 1 4 3 4 3

117 2 1 2 2 4 2

118 4 3 4 4 2 1

119 4 1 4 3 4 3

120 2 3 4 3 4 4

121 2 0 3 1 2 0

122 3 2 2 4 4 3

123 2 3 3 2 4 3

124 3 3 3 3 4 3

125 3 3 1 1 4 4

126 3 2 3 3 4 2

127 4 2 3 4 4 2

128 3 1 3 2 4 1

129 2 2 3 3 3 2

130 3 1 2 4 4 3

131 3 2 2 2 4 3

132 3 1 4 4 2 3

133 4 1 3 3 4 2

134 4 2 4 2 4 4

135 4 3 4 2 4 3

136 4 1 4 1 4 3

137 4 1 3 0 3 3

138 3 0 4 3 3 3

139 2 2 3 2 1 0

140 2 3 4 4 4 4

141 4 2 4 3 4 2

142 1 0 4 2 4 4

143 4 2 4 4 3 3

144 4 4 4 3 3 1

145 4 3 4 3 2 3

146 3 0 1 4 3 3

147 4 4 4 0 4 3

148 3 2 4 2 3 0

149 3 2 4 4 3 2

150 1 4 3 3 3 2

151 3 3 1 4 4 4

152 3 2 4 3 1 2

153 3 2 4 4 4 3

154 1 2 4 1 2 3

155 4 3 4 2 2 3

156 3 0 2 3 4 3

157 3 0 3 1 4 3

158 4 1 4 4 3 4

159 3 0 2 2 3 2

160 2 3 4 2 4 4

161 3 1 3 2 4 4

162 3 3 4 2 3 3

163 4 3 4 2 4 4

164 2 0 3 3 3 0

165 3 2 4 4 4 3

166 2 2 4 0 2 0

[ reached 'max' / getOption("max.print") -- omitted 96 rows ]

> describe(CORA)

Error in describe(CORA) : could not find function "describe"

>

> library(psych)

Attaching package: ‘psych’

The following objects are masked from ‘package:semTools’:

reliability, skew

The following object is masked from ‘package:lavaan’:

cor2cov

> fa.parallel(CORA, fm="ml", fa="fa", n.iter=2000, SMC=TRUE, sim=FALSE,

> quant=0.95, plot=TRUE)

Parallel analysis suggests that the number of factors = 1 and the number of components = NA

>

> library(lavaan)

> ADHS <- 'ADH =~ i1+i2+i3+i4+i5+i6'

> fit1 <- cfa(ADHS, data=CORA, meanstructure=TRUE, estimator="ML",

> std.lv=TRUE) summary(fit1, standardized=TRUE, fit.measures=TRUE,

> modindices=TRUE)

lavaan 0.6-9 ended normally after 15 iterations

Estimator ML

Optimization method NLMINB

Number of model parameters 18

Number of observations 262

Model Test User Model:

Test statistic 37.372

Degrees of freedom 9

P-value (Chi-square) 0.000

Model Test Baseline Model:

Test statistic 775.096

Degrees of freedom 15

P-value 0.000

User Model versus Baseline Model:

Comparative Fit Index (CFI) 0.963

Tucker-Lewis Index (TLI) 0.938

Loglikelihood and Information Criteria:

Loglikelihood user model (H0) -2312.247

Loglikelihood unrestricted model (H1) -2293.561

Akaike (AIC) 4660.494

Bayesian (BIC) 4724.724

Sample-size adjusted Bayesian (BIC) 4667.656

Root Mean Square Error of Approximation:

RMSEA 0.110

90 Percent confidence interval - lower 0.075

90 Percent confidence interval - upper 0.147

P-value RMSEA <= 0.05 0.004

Standardized Root Mean Square Residual:

SRMR 0.032

Parameter Estimates:

Standard errors Standard

Information Expected

Information saturated (h1) model Structured

Latent Variables:

Estimate Std.Err z-value P(>|z|) Std.lv Std.all

ADH =~

i1 1.182 0.072 16.490 0.000 1.182 0.852

i2 0.784 0.066 11.878 0.000 0.784 0.676

i3 1.012 0.078 13.014 0.000 1.012 0.723

i4 0.848 0.074 11.381 0.000 0.848 0.654

i5 0.987 0.075 13.174 0.000 0.987 0.730

i6 1.156 0.075 15.503 0.000 1.156 0.818

Intercepts:

Estimate Std.Err z-value P(>|z|) Std.lv Std.all

.i1 1.840 0.086 21.443 0.000 1.840 1.325

.i2 0.924 0.072 12.890 0.000 0.924 0.796

.i3 2.260 0.086 26.154 0.000 2.260 1.616

.i4 1.916 0.080 23.928 0.000 1.916 1.478

.i5 2.489 0.084 29.776 0.000 2.489 1.840

.i6 1.515 0.087 17.352 0.000 1.515 1.072

ADH 0.000 0.000 0.000

Variances:

Estimate Std.Err z-value P(>|z|) Std.lv Std.all

.i1 0.530 0.068 7.764 0.000 0.530 0.275

.i2 0.731 0.071 10.292 0.000 0.731 0.543

.i3 0.932 0.094 9.931 0.000 0.932 0.477

.i4 0.962 0.092 10.422 0.000 0.962 0.572

.i5 0.855 0.087 9.872 0.000 0.855 0.467

.i6 0.663 0.077 8.620 0.000 0.663 0.332

ADH 1.000 1.000 1.000

Modification Indices:

lhs op rhs mi epc sepc.lv sepc.all sepc.nox

21 i1 ~~ i2 1.901 0.074 0.074 0.119 0.119

22 i1 ~~ i3 4.545 0.136 0.136 0.194 0.194

23 i1 ~~ i4 1.108 0.064 0.064 0.089 0.089

24 i1 ~~ i5 5.602 -0.146 -0.146 -0.217 -0.217

25 i1 ~~ i6 3.040 -0.114 -0.114 -0.193 -0.193

26 i2 ~~ i3 4.552 0.127 0.127 0.154 0.154

27 i2 ~~ i4 0.073 0.016 0.016 0.019 0.019

28 i2 ~~ i5 4.058 -0.115 -0.115 -0.146 -0.146

29 i2 ~~ i6 2.975 -0.096 -0.096 -0.138 -0.138

30 i3 ~~ i4 0.201 -0.030 -0.030 -0.032 -0.032

31 i3 ~~ i5 6.244 -0.166 -0.166 -0.186 -0.186

32 i3 ~~ i6 2.002 -0.093 -0.093 -0.118 -0.118

33 i4 ~~ i5 0.038 0.013 0.013 0.014 0.014

34 i4 ~~ i6 1.240 -0.070 -0.070 -0.088 -0.088

35 i5 ~~ i6 35.325 0.377 0.377 0.501 0.501

>

> ADHS2 <- 'ADH1 =~ i1+i2+i3+i4

+ ADH2 =~ i5+i6'

> fit2 <- cfa(ADHS2, data=CORA, meanstructure=TRUE, estimator="ML",

> std.lv=TRUE) summary(fit2, standardized=TRUE, fit.measures=TRUE,

> modindices=TRUE)

lavaan 0.6-9 ended normally after 20 iterations

Estimator ML

Optimization method NLMINB

Number of model parameters 19

Number of observations 262

Model Test User Model:

Test statistic 4.967

Degrees of freedom 8

P-value (Chi-square) 0.761

Model Test Baseline Model:

Test statistic 775.096

Degrees of freedom 15

P-value 0.000

User Model versus Baseline Model:

Comparative Fit Index (CFI) 1.000

Tucker-Lewis Index (TLI) 1.007

Loglikelihood and Information Criteria:

Loglikelihood user model (H0) -2296.045

Loglikelihood unrestricted model (H1) -2293.561

Akaike (AIC) 4630.090

Bayesian (BIC) 4697.888

Sample-size adjusted Bayesian (BIC) 4637.650

Root Mean Square Error of Approximation:

RMSEA 0.000

90 Percent confidence interval - lower 0.000

90 Percent confidence interval - upper 0.051

P-value RMSEA <= 0.05 0.948

Standardized Root Mean Square Residual:

SRMR 0.014

Parameter Estimates:

Standard errors Standard

Information Expected

Information saturated (h1) model Structured

Latent Variables:

Estimate Std.Err z-value P(>|z|) Std.lv Std.all

ADH1 =~

i1 1.218 0.071 17.055 0.000 1.218 0.877

i2 0.800 0.066 12.143 0.000 0.800 0.690

i3 1.034 0.078 13.327 0.000 1.034 0.739

i4 0.850 0.075 11.365 0.000 0.850 0.656

ADH2 =~

i5 1.055 0.075 14.127 0.000 1.055 0.780

i6 1.274 0.075 17.075 0.000 1.274 0.901

Covariances:

Estimate Std.Err z-value P(>|z|) Std.lv Std.all

ADH1 ~~

ADH2 0.853 0.031 27.165 0.000 0.853 0.853

Intercepts:

Estimate Std.Err z-value P(>|z|) Std.lv Std.all

.i1 1.840 0.086 21.443 0.000 1.840 1.325

.i2 0.924 0.072 12.890 0.000 0.924 0.796

.i3 2.260 0.086 26.154 0.000 2.260 1.616

.i4 1.916 0.080 23.928 0.000 1.916 1.478

.i5 2.489 0.084 29.776 0.000 2.489 1.840

.i6 1.515 0.087 17.352 0.000 1.515 1.072

ADH1 0.000 0.000 0.000

ADH2 0.000 0.000 0.000

Variances:

Estimate Std.Err z-value P(>|z|) Std.lv Std.all

.i1 0.445 0.070 6.362 0.000 0.445 0.231

.i2 0.705 0.070 10.070 0.000 0.705 0.524

.i3 0.887 0.092 9.597 0.000 0.887 0.453

.i4 0.957 0.093 10.311 0.000 0.957 0.570

.i5 0.718 0.084 8.517 0.000 0.718 0.392

.i6 0.375 0.088 4.239 0.000 0.375 0.188

ADH1 1.000 1.000 1.000

ADH2 1.000 1.000 1.000

Modification Indices:

lhs op rhs mi epc sepc.lv sepc.all sepc.nox

26 ADH2 =~ i1 0.188 0.095 0.095 0.069 0.069

27 ADH2 =~ i2 0.576 -0.127 -0.127 -0.109 -0.109

28 ADH2 =~ i3 0.278 -0.106 -0.106 -0.076 -0.076

29 ADH2 =~ i4 0.770 0.166 0.166 0.128 0.128

30 i1 ~~ i2 0.240 -0.029 -0.029 -0.052 -0.052

31 i1 ~~ i3 0.000 0.000 0.000 0.000 0.000

32 i1 ~~ i4 0.001 0.002 0.002 0.003 0.003

33 i1 ~~ i5 0.008 0.005 0.005 0.009 0.009

34 i1 ~~ i6 0.068 0.015 0.015 0.038 0.038

35 i2 ~~ i3 2.193 0.089 0.089 0.112 0.112

36 i2 ~~ i4 0.007 -0.005 -0.005 -0.006 -0.006

37 i2 ~~ i5 0.224 -0.025 -0.025 -0.035 -0.035

38 i2 ~~ i6 0.085 -0.015 -0.015 -0.029 -0.029

39 i3 ~~ i4 0.863 -0.064 -0.064 -0.069 -0.069

40 i3 ~~ i5 0.832 -0.056 -0.056 -0.070 -0.070

41 i3 ~~ i6 0.080 0.017 0.017 0.029 0.029

42 i4 ~~ i5 2.176 0.090 0.090 0.108 0.108

43 i4 ~~ i6 0.150 -0.023 -0.023 -0.038 -0.038

>

> ADHS3 <- 'ADH =~ i1+i2+i3+i4+i5+i6

+ i5 ~~ i6'

> fit3 <- cfa(ADHS3, data=CORA, meanstructure=TRUE, estimator="ML",

> std.lv=TRUE) summary(fit3, standardized=TRUE, fit.measures=TRUE,

> modindices=TRUE)

lavaan 0.6-9 ended normally after 18 iterations

Estimator ML

Optimization method NLMINB

Number of model parameters 19

Number of observations 262

Model Test User Model:

Test statistic 4.967

Degrees of freedom 8

P-value (Chi-square) 0.761

Model Test Baseline Model:

Test statistic 775.096

Degrees of freedom 15

P-value 0.000

User Model versus Baseline Model:

Comparative Fit Index (CFI) 1.000

Tucker-Lewis Index (TLI) 1.007

Loglikelihood and Information Criteria:

Loglikelihood user model (H0) -2296.045

Loglikelihood unrestricted model (H1) -2293.561

Akaike (AIC) 4630.090

Bayesian (BIC) 4697.888

Sample-size adjusted Bayesian (BIC) 4637.650

Root Mean Square Error of Approximation:

RMSEA 0.000

90 Percent confidence interval - lower 0.000

90 Percent confidence interval - upper 0.051

P-value RMSEA <= 0.05 0.948

Standardized Root Mean Square Residual:

SRMR 0.014

Parameter Estimates:

Standard errors Standard

Information Expected

Information saturated (h1) model Structured

Latent Variables:

Estimate Std.Err z-value P(>|z|) Std.lv Std.all

ADH =~

i1 1.218 0.071 17.055 0.000 1.218 0.877

i2 0.800 0.066 12.143 0.000 0.800 0.690

i3 1.034 0.078 13.327 0.000 1.034 0.739

i4 0.850 0.075 11.365 0.000 0.850 0.656

i5 0.900 0.078 11.477 0.000 0.900 0.665

i6 1.087 0.077 14.045 0.000 1.087 0.769

Covariances:

Estimate Std.Err z-value P(>|z|) Std.lv Std.all

.i5 ~~

.i6 0.366 0.075 4.869 0.000 0.366 0.401

Intercepts:

Estimate Std.Err z-value P(>|z|) Std.lv Std.all

.i1 1.840 0.086 21.443 0.000 1.840 1.325

.i2 0.924 0.072 12.890 0.000 0.924 0.796

.i3 2.260 0.086 26.154 0.000 2.260 1.616

.i4 1.916 0.080 23.928 0.000 1.916 1.478

.i5 2.489 0.084 29.776 0.000 2.489 1.840

.i6 1.515 0.087 17.352 0.000 1.515 1.072

ADH 0.000 0.000 0.000

Variances:

Estimate Std.Err z-value P(>|z|) Std.lv Std.all

.i1 0.445 0.070 6.362 0.000 0.445 0.231

.i2 0.705 0.070 10.070 0.000 0.705 0.524

.i3 0.887 0.092 9.597 0.000 0.887 0.453

.i4 0.957 0.093 10.311 0.000 0.957 0.570

.i5 1.021 0.101 10.087 0.000 1.021 0.558

.i6 0.817 0.089 9.131 0.000 0.817 0.409

ADH 1.000 1.000 1.000

Modification Indices:

lhs op rhs mi epc sepc.lv sepc.all sepc.nox

22 i1 ~~ i2 0.240 -0.029 -0.029 -0.052 -0.052

23 i1 ~~ i3 0.000 0.000 0.000 0.000 0.000

24 i1 ~~ i4 0.001 0.002 0.002 0.003 0.003

25 i1 ~~ i5 0.008 0.005 0.005 0.008 0.008

26 i1 ~~ i6 0.068 0.015 0.015 0.026 0.026

27 i2 ~~ i3 2.193 0.089 0.089 0.112 0.112

28 i2 ~~ i4 0.007 -0.005 -0.005 -0.006 -0.006

29 i2 ~~ i5 0.224 -0.025 -0.025 -0.030 -0.030

30 i2 ~~ i6 0.085 -0.015 -0.015 -0.020 -0.020

31 i3 ~~ i4 0.863 -0.064 -0.064 -0.069 -0.069

32 i3 ~~ i5 0.832 -0.056 -0.056 -0.059 -0.059

33 i3 ~~ i6 0.080 0.017 0.017 0.020 0.020

34 i4 ~~ i5 2.176 0.090 0.090 0.091 0.091

35 i4 ~~ i6 0.150 -0.023 -0.023 -0.026 -0.026

>

> library(semTools)

> tabelle <- compareFit(fit1, fit2, fit3, nested = FALSE)

> summary(tabelle)

####################### Model Fit Indices ###########################

chisq df pvalue rmsea cfi tli srmr aic bic

fit2 4.967† 8 .761 .000† 1.000† 1.007† .014 4630.090† 4697.888†

fit3 4.967 8 .761 .000† 1.000† 1.007 .014† 4630.090 4697.888

fit1 37.372 9 .000 .110 .963 .938 .032 4660.494 4724.724

>

> ## Paket ezCutoffs laden

> library(ezCutoffs)

ezCutoffs is in development. Please report any bugs and check <https://github.com/bschmalbach/ezCutoffs> for newer versions.

Attaching package: ‘ezCutoffs’

The following object is masked from ‘package:semTools’:

compareFit

> ## Eingabe von lavvan Objekt, Datenobjekt, Anzahl der Replikationen,

> Fit-Indizes, Schätzfunktion, alpha Level und Kernanzahl out <-

> ezCutoffs(ADHS2, CORA, n_rep = 10000, fit_indices = c("cfi", "rmsea",

> "srmr"), estimator = "ml",normality="empirical", alpha_level =.05,

> n_cores = 1)

Data Generation

|==================================================| 100% elapsed = 1m ~ 0s

Model Fitting

|==================================================| 100% elapsed = 3m ~ 0s Warning messages:

1: In lav_object_post_check(object) :

lavaan WARNING: some estimated ov variances are negative

2: In lav_object_post_check(object) :

lavaan WARNING: some estimated ov variances are negative

> summary(out)

#Runs #Converged Estimator Alpha TotalObservations Missing

10000 10000 ml 0.05 262 listwise

Empirical fit Simulation Mean Simulation SD Simulation Median Cutoff (alpha = 0.05)

cfi 1.00000000 0.99743374 0.004158702 1.0000000 0.98879087

rmsea 0.00000000 0.01941873 0.023885643 0.0000000 0.06465712

srmr 0.01588972 0.01779940 0.004828843 0.0175098 0.02617207

> plot(out)

`stat_bin()` using `bins = 30`. Pick better value with `binwidth`.
